# Thyroid and Its Ripple Effect: Impact on Cardiac Structure, Function, and Outcomes

**DOI:** 10.7759/cureus.51574

**Published:** 2024-01-03

**Authors:** Olusegun A Olanrewaju, Rida Asghar, Sameeta Makwana, Muhammad Yahya, Narendar Kumar, Muhammad Hasnain Khawar, Aqsa Ahmed, Tamur Islam, Komal Kumari, Sujeet Shadmani, Mohsin Ali, Satesh Kumar, Mahima Khatri, Giustino Varrassi, Tamam Mohamad

**Affiliations:** 1 Pure and Applied Biology, Ladoke Akintola University of Technology, Ogbomoso, NGA; 2 General Medicine, Stavropol State Medical University, Stavropol, RUS; 3 Medicine, Lahore Medical and Dental College, Lahore, PAK; 4 Medicine, Jinnah Sindh Medical University, Karachi, PAK; 5 Medicine, Liaquat University of Medical and Health Sciences, Jamshoro, PAK; 6 Internal Medicine, Burjeel Hospital, Abu Dhabi, ARE; 7 Internal Medicine, Mayo Hospital, Lahore, PAK; 8 Medicine, Medicare Hospital, Faisalabad, PAK; 9 Internal Medicine, Allied Hospital, Faisalabad, PAK; 10 Medicine, NMC Royal Family Medical Centre, Abu Dhabi, ARE; 11 Medicine, Shaheed Mohtarma Benazir Bhutto Medical College, Karachi, PAK; 12 Medicine, Shaheed Mohtarma Benazir Bhutto Medical University, Larkana, PAK; 13 Internal Medicine/Cardiology, Dow University of Health Sciences, Karachi, PAK; 14 Pain Medicine, Paolo Procacci Foundation, Rome, ITA; 15 Cardiovascular Medicine, Wayne State University, Detroit, USA

**Keywords:** molecular mechanisms, endocrine disorders, cardiovascular outcomes, thyroid-cardiac interplay, cardiac manifestations, thyroid dysfunction

## Abstract

Thyroid dysfunction is a widespread and complex issue in the field of endocrine disorders. It has a significant impact on multiple physiological systems, particularly on the heart. This review explores the complex interaction between thyroid dysfunction and cardiac dynamics, examining the detailed network of molecular, cellular, and systemic changes that underlie the close connection between these two physiological areas. Thyroid dysfunction, which includes both hyperthyroidism and hypothyroidism, is a common endocrine condition that affects millions of people worldwide. The thyroid hormones thyroxine and triiodothyronine regulate various metabolic activities essential for maintaining cellular balance. Disruptions in thyroid function result in widespread consequences, affecting the cardiovascular system. Thyroid hormones directly impact cardiac muscle cells, controlling their ability to contract, their electrical properties, and their reaction to hypertrophy. Thyroid dysfunction goes beyond the level of individual cells and involves complex interactions among vascular dynamics, neurohormonal control, and endothelial function. These factors all contribute to the development of cardiovascular illness. The impact of thyroid dysfunction on cardiac structure, function, and outcomes is not limited to a one-way pattern. Instead, it involves a dynamic two-way interaction. The manifestations of this condition can vary from minor changes in the electrical activity of the heart to more obvious structural abnormalities, such as an increase in the size of the heart muscle and a decrease in its ability to relax during the filling phase. Furthermore, the correlation between thyroid dysfunction and adverse cardiovascular outcomes, such as heart failure and arrhythmias, highlights the clinical importance of this connection. This review provides a complete overview of the relationship between thyroid dysfunction and cardiac dynamics by analyzing a wide range of research from clinical, molecular, and epidemiological perspectives. This study seeks to enhance our comprehension of the comprehensive effects of thyroid dysfunction on the anatomy and function of the heart by explaining the complex molecular mechanisms and systemic consequences. The goal is to establish a basis for informed clinical treatment and future research efforts.

## Introduction and background

The complex coordination of hormone production and regulation in the human body is a fragile balance that, when disturbed, can have widespread effects on various physiological systems. Thyroid dysfunction is a complex and widespread difficulty among various endocrine illnesses. The thyroid gland in the front of the neck is the main source of thyroid hormone production, releasing thyroxine (T4) and triiodothyronine (T3) into the bloodstream. Thyroid hormones, which are molecular messengers, significantly impact cellular metabolism, growth, and differentiation [1]. Thyroid dysfunction presents itself in two main forms: hypothyroidism, which is defined by inadequate hormone production, and hyperthyroidism, which is characterized by an excessive amount of hormone production. Both states, referring to hypo and hyperthyroidism, disturb the delicate equilibrium necessary for maintaining cellular homeostasis, resulting in widespread effects that go beyond the thyroid gland. Considering the widespread impact of thyroid hormones, it is unsurprising that thyroid dysfunction affects various organ systems [1]. Nevertheless, the influence on the cardiovascular system is especially remarkable, demonstrating an intricate interaction encompassing various levels, from the molecular to the clinical domains. Thyroid hormones directly impact cardiac myocytes in the cardiovascular field, affecting their contractility, electrophysiology, and hypertrophic responses. The intricate control of the vasculature is also influenced by them, affecting vascular dynamics, neurohormonal regulation, and endothelial function.

Therefore, thyroid dysfunction plays a crucial role in the development of cardiovascular disease. Comprehending the subtle complexities of thyroid disease is essential for unraveling its extensive impact on the heart's structure, function, and clinical results. To understand the complex relationship between thyroid dysfunction and the functioning of the heart, it is crucial to explore the deep importance of thyroid hormones in the body's physiological processes. The primary thyroid hormones, T3 and T4, control cellular metabolism by adjusting gene expression. Their impacts are facilitated by attaching to nuclear receptors called thyroid hormone receptors (THR), which are widely present in many organs, including the heart. The subsequent biochemical sequence triggered by the binding of hormones to receptors coordinates a series of processes that affect cellular activity [2]. Thyroid hormones impact the cardiovascular system, not just in the heart muscle but also in regulating blood vessel function. Thyroid hormones significantly impact heart rate, contractility, and blood pressure and are crucial for maintaining cardiovascular balance. Furthermore, they play a role in controlling lipid metabolism, impacting cholesterol levels and the development of atherosclerosis. The precise equilibrium of thyroid hormones is essential for maintaining cardiovascular health [2]. The global incidence of thyroid dysfunction highlights its relevance as a public health concern. Epidemiological studies have identified diverse prevalence rates of thyroid dysfunction among distinct communities and age cohorts. An extensive examination of thyroid function in the United States revealed a range of hypothyroidism prevalence rates that were influenced by age and gender. The study revealed a greater occurrence of hypothyroidism in women, namely in those aged 60 and above, with an overall prevalence rate of 4.6% [3]. Similarly, the occurrence of hyperthyroidism has been recorded with different rates of prevalence. Research has indicated that the occurrence of hyperthyroidism varies between 0.5% and 2%. The results highlight the extensive influence of thyroid dysfunction, underscoring the need to thoroughly investigate its effects, especially in areas such as cardiovascular well-being [4]. The clinical consequences of thyroid dysfunction go beyond hormonal imbalances, with the cardiovascular system being a primary focus. The reciprocal correlation between thyroid disease and heart dynamics has gained growing recognition in clinical literature. Thyroid dysfunction, whether it is underactive (hypothyroidism) or overactive (hyperthyroidism), is linked to a range of cardiovascular effects. These effects can vary from minor changes in the heart's electrical activity, as shown on an electrocardiogram (ECG), to more obvious structural changes in the heart. The effect on the structure and function of the heart is significant, including an increase in the heart muscle's size, reduced heart's ability to relax, and changes in its ability to contract. Moreover, thyroid dysfunction has been linked to the development of cardiovascular disorders such as heart failure and arrhythmias, exerting a major impact on patient outcomes [5].

Ascertaining the clinical importance of thyroid-cardiac interactions is, thus, crucial for expanding our understanding of the comprehensive consequences of thyroid dysfunction. An examination of thyroid dysfunction and the importance of thyroid hormones in the body's functioning lays the foundation for a thorough investigation into the frequency of thyroid malfunction and the clinical implications of the relationship between the thyroid and the heart. This study aims to explore the molecular mechanisms, structural alterations, functional implications, and clinical manifestations that underlie the impact of the thyroid on heart structure, function, and outcomes. This study aims to enhance the current understanding of the crucial confluence of endocrinology and cardiology by thoroughly examining available research.

## Review

Methodology

This review employs a rigorous and systematic approach to thoroughly examine the complex relationship between thyroid dysfunction and its impact on the heart's anatomy, function, and clinical outcomes. A comprehensive literature review was performed on prominent databases, including PubMed/MEDLINE, Embase, Cochrane Library, and Scopus, using a specific set of search terms such as "thyroid dysfunction," "cardiovascular consequences," "thyroid hormones and heart," "cardiac structure and thyroid dysfunction," and "thyroid dysfunction and cardiovascular outcomes." The inclusion criteria consisted of publications published in English from 2000 onwards, guaranteeing a modern viewpoint on the topic. The analysis encompassed human and animal studies, specifically examining the molecular, structural, and clinical aspects of the interactions between the thyroid and the heart. Nevertheless, it is crucial to acknowledge specific constraints inherent in this evaluation. First, the extensive nature of the subject may introduce a selection bias in the article choices, and despite thorough search tactics, some pertinent research may unintentionally be omitted. In addition, the review heavily depends on existing literature. Although attempts are made to incorporate a wide range of studies, it is only possible to eliminate the potential for publication bias partially. Utilizing a thematic synthesis strategy facilitates the amalgamation of research outcomes from several investigations, resulting in a coherent and cohesive narrative of the subject matter. It is crucial to note that ethical committee permission was not obtained for this narrative review. Due to its nature as a compilation of existing material, ethics committee approval was considered unnecessary rather than involving collecting primary data from human beings or animals. However, the review maintains ethical standards by accurately citing sources and disclosing the techniques and limits of this narrative synthesis.

Thyroid hormones and cardiovascular system

The thyroid hormones, specifically T4 and T3, intricately regulate the circulatory system, which consists of a complex network of structures and functions. This section explores the process of creating and controlling thyroid hormones, their movement and absorption into cells, and the existence of thyroid receptors in the circulatory system. It reveals the complex mechanisms by which thyroid hormones exert their significant impact.

Synthesis and Regulation of Thyroid Hormones

Thyroid hormones are synthesized in the thyroid gland, an organ shaped like a butterfly in the neck. The process initiates with the absorption of iodide from the circulatory system by thyroid follicular cells. The iodide is later integrated into tyrosine residues in thyroglobulin, a glycoprotein in thyroid follicles' colloids. Thyroid peroxidase, an enzyme, speeds up adding iodine to tyrosine residues and helps connect this iodinated tyrosine to create T4 and T3 hormones [5]. The process of thyroid hormone synthesis is closely regulated and principally controlled by the hypothalamus-pituitary-thyroid axis. The hypothalamus releases thyrotropin-releasing hormone, which prompts the anterior pituitary gland to secrete thyroid-stimulating hormone (TSH). TSH stimulates the thyroid gland to secrete and release T4 and T3. This complex feedback mechanism guarantees to regulate thyroid hormone levels within a precise physiological range [5].

Transport and Uptake of Thyroid Hormones by Cells

After being created, T4 and T3 are released into the bloodstream and travel while attached to plasma proteins, mainly thyroxine-binding globulin, albumin, and transthyretin. The carrier proteins function as storage units, controlling the supply of thyroid hormones to specific tissues. Although T4 is the main form released by the thyroid gland, T3, which is more physiologically active, is produced by converting T4 to T3 in other tissues, such as the liver and kidneys [6]. Thyroid hormones are taken up by cells by specialized transporters, including the monocarboxylate transporter and organic anion-transporting polypeptides. These transporters facilitate the entry of T4 and T3 into target cells, enabling them to exercise their effects on cellular function. The importance of cellular absorption resides in the tissue-specific transformation of T4 into T3, which adjusts the local level of the active hormone according to physiological needs [6].

Thyroid Receptors in the Cardiovascular System

Thyroid hormones exert their biological effects by interacting with THR, which belong to the nuclear receptor superfamily. There are two main isoforms of THR, namely thyroid hormone receptor alpha (THRα) and thyroid hormone receptor beta (THRβ), which have different tissue distribution and activities. Both isoforms are present in cardiac myocytes, vascular smooth muscle cells, and endothelial cells within the cardiovascular system [6]. The activation of THR occurs when thyroid hormones bind to the ligand-binding domain of the receptor, causing structural modifications that allow the receptor to form a heterodimer with the retinoid X receptor (RXR). This intricate molecule moves into the nucleus and attaches itself to specific sections called thyroid hormone response elements (TREs) located within the promoter regions of target genes. By doing so, it regulates the activity of these genes by affecting their transcription process. Thyroid hormones have a crucial role in the heart by affecting gene expression, essential for cardiac development, contractility, and electrophysiology. Thyroid hormones increase the activity of genes that have a role in the strength of the heart's contractions, such as sarcoendoplasmic reticulum calcium transport ATPase (SERCA) and myosin heavy chain (MHC) [7]. In addition, they have regulatory effects on ion channels, which contribute to the modification of cardiac electrophysiology. The vascular effects of thyroid hormones are similarly significant. Thyroid hormones influence the expression of genes involved in regulating vascular tone, such as endothelial nitric oxide synthase (eNOS) and angiotensin-converting enzyme (ACE), specifically inside the cells of vascular smooth muscle. Furthermore, thyroid hormones directly influence endothelial cells, affecting the synthesis of NO and the function of the endothelium [7]. To summarize, the existence of thyroid receptors in the cardiovascular system highlights the close connection between thyroid hormones and the functioning of the heart. The activation of THRα and THRβ triggers a series of biochemical processes that regulate gene expression in the heart and blood vessels, hence playing a crucial role in precisely controlling cardiovascular function. The complex interaction between thyroid hormones and cardiovascular homeostasis underscores their essential role and the importance of comprehending their molecular mechanisms about heart shape and function. Table 1 shows an overview of thyroid hormones and cardiac receptors in great detail.

**Table 1:** Overview of thyroid hormones and cardiac receptors T3: Triiodothyronine; T4: Thyroxine

Parameter	Thyroid hormones (T3, T4) in the heart
Thyroid hormones	Triiodothyronine (T3) and thyroxine (T4)
Receptors in the heart	Thyroid hormone receptors (THR), mainly TRα and TRβ
Location of receptors	Present in cardiac myocytes (muscle cells of the heart)
Binding affinity	Higher affinity for T3 compared to T4
Influence on gene expression	Activates or suppresses genes involved in cardiac development and function
Cardiac structure effects	Promotes cardiac hypertrophy (increase in size of heart cells), affects myocardial contractility, influences angiogenesis (formation of new blood vessels in the heart)
Cardiac function effects	Modulates heart rate and rhythm, alters blood pressure regulation, impacts cardiac output and stroke volume
Electrophysiological effects	Alters ion channel activity, affecting action potential, influences electrical conduction in the heart
Overall impact on the heart	Thyroid hormones play a crucial role in maintaining cardiac homeostasis, dysregulation can lead to cardiac dysfunction and heart diseases

Molecular mechanisms

Thyroid Hormone Signaling Pathways in Cardiac Cells

The heart's cellular reactions to thyroid hormones are precisely regulated by a complex network of signaling channels that convert the hormonal signals into distinct physiological consequences. The THR, particularly THRα and THRβ, are the primary agents responsible for mediating the effects of thyroid hormone. These receptors are highly expressed in cardiac myocytes [7]. The conventional genomic signaling pathway entails the interaction between thyroid hormones and THR, forming a complex with the RXR. This intricate molecule moves to the nucleus and attaches itself to specific regions called TREs in the promoter regions of target genes. This action triggers the transcription process and subsequently leads to various cellular responses. Moreover, non-genomic signaling pathways play a role in the swift impacts of thyroid hormones on cardiac cells. These pathways encompass membrane receptors, specifically integrin αvβ3, facilitating intracellular signaling events via kinase cascades. Activation of these pathways can result in swift alterations in ion flow, contractility, and cellular metabolism. The interaction between genetic and non-genomic signaling pathways guarantees a dynamic and subtle reaction to thyroid hormones in cardiac cells [8].

Impact on Cardiac Gene Expression

The regulation of gene expression by thyroid hormones is a fundamental aspect of their impact on heart physiology. Thyroid hormones control the activity of several genes involved in the heart's formation, contraction, and electrical activity. THR regulate the expression of essential genes involved in excitation-contraction coupling in cardiac myocytes [8]. An important objective is the SERCA, which is accountable for calcium uptake back into the sarcoplasmic reticulum. Thyroid hormones stimulate SERCA's expression, which improves calcium management and leads to enhanced contractility. Furthermore, thyroid hormones regulate the production of MHC isoforms, affecting heart muscle contractile characteristics [8]. In addition to excitation-contraction coupling, thyroid hormones have regulatory effects on ion channels, influencing the shape of the cardiac action potential. Thyroid hormones intricately regulate the expression of potassium, sodium, and calcium channels, which in turn affect the duration and amplitude of the action potential. The molecular modifications mentioned here play a role in the overall electrophysiological effects of thyroid hormones on the heart. Moreover, thyroid hormones influence genes linked to heart hypertrophy and remodeling. The rise in expression of hypertrophic markers, specifically atrial natriuretic peptide (ANP) and brain natriuretic peptide (BNP), demonstrates the involvement of thyroid hormones in coordinating structural modifications in response to heightened workload [9]. The complex control of gene expression by thyroid hormones in cardiac cells emphasizes their central role in creating the molecular composition of the heart.

Cellular Processes Modulated by Thyroid Hormones

Thyroid hormones broadly impact cellular processes essential for the proper functioning of the heart, going beyond only regulating gene expression. A necessary consequence is the stimulation of cellular metabolism, which leads to higher energy production and consumption. Thyroid hormones enhance the function of essential enzymes in the glycolytic and oxidative phosphorylation pathways, promoting ATP synthesis to fulfill the hypermetabolic state's increased energy requirements [10]. Thyroid hormones are involved in metabolic control and significantly impact the formation of new blood vessels (angiogenesis) and the restructuring of blood vessels (vascular remodeling) in the heart tissue. Thyroid hormones stimulate the production of vascular endothelial growth factor and fibroblast growth factor, encouraging the development of new blood vessels. This ensures that the myocardium, which has increased metabolic activity, receives sufficient oxygen and nutrients. The interaction between thyroid hormones and cellular death is an additional aspect of their impact on cardiac cells. Short-term exposure to thyroid hormones may have a protective effect against cell death, but long-term increases might cause cell death, which can contribute to changes in the structure and function of the heart [10]. The complex equilibrium between signals that promote cell survival and signals that promote programmed cell death highlights the context-specific impact of thyroid hormones on cellular viability. Furthermore, thyroid hormones regulate cellular adhesion and migration, impacting the extracellular matrix's (ECM's) restructuring. Thyroid hormones control the levels of matrix metalloproteinases (MMPs) and tissue inhibitors of metalloproteinases (TIMPs). This regulation is essential for the continuous remodeling of the cardiac matrix, which is necessary for tissue repair and adaptability to different physiological situations [11]. To summarize, the molecular processes that govern the effects of thyroid hormones in cardiac cells are varied and complex. The substantial influence of thyroid hormones on heart physiology is attributed to integrating genomic and non-genomic communication pathways, the precise regulation of gene expression, and the modulation of cellular processes. Gaining a comprehensive understanding of these complicated molecular pathways is crucial for deciphering the intricacies of the interactions between the thyroid and the heart. It also provides valuable insights into possible therapeutic approaches for cardiovascular disorders linked to thyroid dysfunction.

Structural changes in the heart

The significant impact of thyroid hormones on the structural integrity and function of the heart is demonstrated by a wide range of alterations, including hypertrophy, remodeling of cardiac chambers, and the formation of fibrosis. This section thoroughly examines every aspect, revealing the complex anatomical changes caused by thyroid disease.

Thyroid-Induced Cardiac Hypertrophy

Cardiac hypertrophy, an enlargement of individual cardiomyocytes, is a prominent structural alteration caused by thyroid insufficiency. Thyroid hormones, including T3, have a crucial function in controlling the heart's formation through genetic and non-genomic routes. Stimulation of THR in heart muscle cells initiates a series of chemical reactions that result in hypertrophic responses [11]. Thyroid hormones primarily induce hypertrophic effects by increasing the activity of crucial signaling pathways involved in cellular growth and protein synthesis. The protein kinase B/mammalian target of rapamycin (Akt/mTOR) pathway is vital in mediating thyroid-induced heart hypertrophy. Stimulation of this route enhances the production of proteins and hinders the breakdown of proteins, resulting in the expansion of cardiomyocytes [11]. Furthermore, thyroid hormones impact the manifestation of hypertrophic indicators, such as ANP and BNP. These peptides, typically linked to abnormal heart enlargement, are increased in response to thyroid hormones, suggesting the intricate nature of structural alterations caused by the thyroid. The heart tissue undergoes hypertrophic remodeling due to thyroid dysfunction, highlighting the delicate equilibrium between physiological adaptation and maladaptive reactions.

Remodeling of Cardiac Chambers

Thyroid dysfunction, along with hypertrophy, causes changes in the structure and shape of the heart, affecting its overall architecture and geometry. The ventricular chambers experience dimensions, morphology, and myocardial thickness modifications, which impact heart performance. The influence of thyroid hormones on chamber remodeling is complex, encompassing genetic and non-genomic processes [12]. Thyroid hormones influence the expression of genes related to the remodeling of the ECM, hence impacting the synthesis and breakdown of collagen. This alteration of ECM dynamics has a role in modifying myocardial stiffness and compliance. Thyroid hormones have a collagenolytic effect on the cardiac interstitium, which MMPs facilitate. This leads to additional remodeling of the cardiac interstitium [13].

Furthermore, the non-genomic impacts of thyroid hormones on ion channels and calcium regulation lead to changes in the ability of the heart to contract and relax, affecting the mechanical characteristics of the heart muscle. The combination of functional adaptations and structural alterations contributes to the overall remodeling of heart chambers. The effect of thyroid-induced chamber remodeling varies among the different heart chambers. Although hypertrophy and dilatation can occur in both ventricles, the left ventricle tends to undergo more significant changes because of its involvement in systemic circulation. The chamber-specific modifications highlight the spatial diversity of structural remodeling caused by thyroid activity [13].

Fibrosis and Thyroid Dysfunction

The presence of thyroid dysfunction is closely associated with fibrosis in the myocardium, which is a significant structural change that dramatically impacts cardiac function. Fibrosis, a condition marked by the excessive accumulation of collagen and other components of the ECM, causes a disturbance in the typical structure of the heart tissue. This disruption results in reduced contractility and heightened stiffness [14]. Thyroid hormones play a role in regulating fibrosis through intricate interactions between signaling pathways that promote fibrosis and those that inhibit it. Thyroid hormones stimulate the upregulation of transforming growth factor-beta (TGF-β), a powerful promoter of fibrosis. TGF-β activation triggers a series of processes that transform fibroblasts into myofibroblasts, which play a crucial role in producing and depositing collagen. In contrast, thyroid hormones also have anti-fibrotic effects by influencing factors such as MMPs and TIMPs. The degree of fibrosis in the heart affected by thyroid dysregulation is determined by the equilibrium between these variables that promote and inhibit fibrosis [14]. Fibrosis in thyroid dysfunction occurs in nature and spreads to the perivascular areas. Perivascular fibrosis has a role in changes to blood flow in the coronary arteries and can worsen the adverse effects of structural changes caused by thyroid-related issues. To summarize, thyroid dysfunction leads to many changes in the heart's structure, including hypertrophy, remodeling of the cardiac chambers, and the formation of fibrosis. The complex relationship between genomic and non-genomic pathways, along with the careful equilibrium between beneficial and harmful responses, highlights the complicated character of these structural changes [15]. Comprehending these alterations is essential for deciphering the pathophysiological effects of thyroid dysfunction on the structure and function of the heart.

Functional consequences

The thyroid gland's malfunction significantly affects the heart's functional behavior, resulting in impaired contraction and relaxation. This section thoroughly examines the practical outcomes, analyzing the changes in the heart muscle's ability to contract, the heart's electrical activity, the power of nature to relax, and its compliance, as well as the implications for diastolic heart failure (DHF).

Contractile Dysfunction

The complex correlation between thyroid hormones and the ability of the heart muscles to contract is crucial in understanding the functional effects of thyroid disorders. Thyroid hormones, specifically T3, are vital in controlling the activation of genes related to excitation-contraction coupling. This directly affects the ability of cardiac myocytes to contract effectively. Thyroid hormones increase the expression of SERCA, which improves the uptake of calcium into the sarcoplasmic reticulum [15]. This, in turn, boosts the efficiency of cardiac contraction. In addition, the adjustment of ion channels, such as the sodium-potassium pump and L-type calcium channels, precisely regulates the balance of ions within cardiac muscle cells, affecting contractility. Nevertheless, the intricate equilibrium necessary for ideal contractile performance can be disturbed in the context of thyroid disease. Hypothyroidism, marked by diminished thyroid hormone levels, results in a decline in contractility due to compromised calcium regulation and decreased expression of contractile proteins. On the other hand, hyperthyroidism, characterized by high levels of thyroid hormones, can lead to increased heart muscle contraction, which may make the heart more susceptible to disorders like atrial fibrillation (AF) and hypertrophic cardiomyopathy [16]. The physiological effects of changes in myocardial contractility caused by the thyroid extend beyond the cellular level and affect the overall functionality of the heart. Modifications in contractility impact stroke volume, ejection fraction, and cardiac output, thus molding the hemodynamic characteristics of the heart. Hence, a comprehensive comprehension of the molecular pathways that cause alterations in contractility due to the thyroid is necessary to decipher the impact on cardiac physiology. Thyroid hormones carefully regulate the heart's electrical activity, affecting the duration of action potentials, the speed of conduction, and the intervals of recovery. The changes in electrophysiological parameters have significant consequences for the vulnerability to arrhythmias and the overall electrical stability of the heart [17]. Thyroid hormones impact the development and operation of ion channels that regulate the cardiac action potential. Thyroid hormones increase the potassium channel activity, which decreases the length of action potentials and promotes fast repolarization. In contrast, the impact on sodium and calcium channels directly affects depolarization, which influences the initiation and propagation of action potentials. Thyroid-induced changes in ion channel function ultimately led to a modification in the electrocardiographic profile. Hyperthyroidism is a condition where there are higher levels of thyroid hormones. This condition is linked to a shorter QT interval, which indicates that the ventricular myocardium repolarizes faster. This makes patients more susceptible to diseases such as AF and ventricular tachycardia. In contrast, hypothyroidism is associated with an extended QT interval, which indicates a delay in the repolarization of the ventricles. Comprehensive restoration of the heart's electrical potential increases susceptibility to torsades de pointes, a type of irregular, rapid heartbeat in the heart's lower chambers, which is linked to a higher likelihood of sudden cardiac arrest. The alterations in cardiac electrophysiology caused by the thyroid emphasize the delicate equilibrium necessary to sustain electrical stability in the heart [18].

Diastolic Dysfunction

Diastolic dysfunction, which refers to poor relaxation and increased stiffness during the diastolic part of the heart cycle, is a specific result of thyroid dysfunction. The effect on diastolic function is complex, encompassing changes in the elasticity of the heart muscle, the speed of relaxation, and the movement of calcium inside the cells. Thyroid hormones impact the activation of genes associated with the flexibility of the heart muscle, namely those involved in the restructuring of the material surrounding the cells (known as extracellular matrix or ECM). Thyroid-induced activation of TGF-β leads to increased collagen synthesis, resulting in elevated cardiac stiffness. This increased rigidity hinders the relaxation of the ventricles during diastole, resulting in less filling of the ventricles [18]. In addition, thyroid hormones also impact the diastolic phase by affecting the process of calcium reuptake into the sarcoplasmic reticulum. Modified calcium regulation delays relaxation, resulting in a more extended period of isovolumic peace and hindering the initial filling stage of the cardiac cycle. The combined effect of thyroid-related diastolic dysfunction has significant consequences for the progression of DHF. DHF, which is marked by symptoms of heart failure without apparent systolic dysfunction, is strongly linked to diseases like hypothyroidism and subclinical hyperthyroidism. Hypothyroidism leads to heightened myocardial stiffness and poor relaxation, which in turn contribute to raised left ventricular filling pressures. These factors make persons more likely to experience symptoms such as difficulty breathing, reduced ability to exercise, and accumulation of fluid in the lungs - characteristic signs of DHF. The connection between hypothyroidism and DHF highlights the importance of thyroid-related changes in diastolic function in a clinical context [19]. On the other hand, subclinical hyperthyroidism, which is defined by slightly increased levels of thyroid hormones, is also associated with the occurrence of diastolic dysfunction. The increased strength of the heart's contractions that occurs in hyperthyroidism, along with changes in how calcium is managed and the remodeling of the ECM, leads to problems with the heart's ability to relax during diastole. The acknowledgment of diastolic dysfunction as a possible outcome of thyroid dysfunction emphasizes the importance of careful monitoring and prompt intervention to prevent the development of symptomatic heart failure. To summarize, thyroid disease has intricated and diverse effects on the heart, impacting contractility and diastolic function. The complex interaction of thyroid hormones, ion channels, and structural elements of nature determines the overall functional characteristics of the myocardial. It is essential to thoroughly comprehend these functional outcomes to identify the pathophysiological mechanisms that cause thyroid-induced heart dysfunction and develop specific therapeutic approaches.

Thyroid dysfunction and vascular dynamics

The complex interaction between thyroid function and circulatory dynamics is a diverse and crucial part of cardiovascular physiology. This section explores the intricate relationship between thyroid dysfunction and its impact on endothelial function, vascular resistance, blood pressure management, and the development of atherosclerosis. It provides a thorough understanding of the linked pathways that influence cardiovascular health.

Endothelial Function

The state of the endothelium, regulated by the precise equilibrium of chemicals that affect blood vessel constriction and dilation, plays a crucial role in maintaining the vascular system's health. Thyroid hormones, including T3, substantially impact endothelial function through genetic and non-genomic processes. Thyroid hormones are vital in controlling the synthesis of nitric oxide (NO) in the endothelium. NO, a potent vasodilator, is crucial in maintaining vascular tone and controlling blood flow. Studies have demonstrated that T3 increases the production and effectiveness of eNOS, the enzyme responsible for synthesizing NO [19]. This increase enhances the process of vasodilation and guarantees optimal endothelial function. In contrast, thyroid dysfunction can disturb the equilibrium between signals that widen blood vessels and signals that narrow blood vessels. Hypothyroidism is linked to increased levels of endothelin-1, a substance that narrows blood vessels, and decreased availability of NO, leading to impaired function of the inner lining of blood vessels. The imbalance of these vasoactive chemicals leads to changes in blood vessel constriction and a decrease in the ability of the endothelium to widen blood vessels [19]. Thyroid dysfunction regulates inflammatory and oxidative stress pathways in the vascular endothelium. Hypothyroidism, which is defined by decreased levels of thyroid hormones, is linked to elevated levels of pro-inflammatory cytokines and signs of oxidative stress. The presence of inflammation in the body disrupts the delicate equilibrium of vasoactive chemicals, leading to endothelial dysfunction [20]. Comprehending the subtle distinctions in how thyroid hormones affect endothelial function is crucial for deciphering the complex connection between thyroid disorders and vascular dynamics.

Vascular Resistance and Blood Pressure Regulation

Thyroid function is closely linked to regulating vascular resistance and blood pressure. Thyroid hormones significantly impact the smooth muscle in blood vessels, affecting the constriction of vessels and the overall blood pressure in the body through multiple pathways. Thyroid hormones influence the sympathetic nervous system (SNS), crucial in regulating blood pressure. Hyperthyroidism is linked to elevated SNS activity, resulting in heightened vascular constriction and higher heart output. Excessive adrenergic activity leads to increased blood pressure levels, highlighting the direct influence of thyroid dysfunction on the resistance of blood vessels [20]. Thyroid hormones also affect the renin-angiotensin-aldosterone system, essential for regulating blood pressure. Hyperthyroidism is linked to elevated levels of renin and aldosterone, which stimulate vasoconstriction and the retention of salt. These hormonal fluctuations result in heightened vascular resistance and raised blood pressure. The interaction between endothelin and NO, as mentioned in the context of endothelial function, also affects the regulation of vascular resistance. Disruptions in the levels of NO and endothelin, which are frequently seen in thyroid dysfunction, play a role in changing vascular tone and resistance. The dysregulation highlights the intricate nature of the thyroid's impact on controlling blood pressure [21].

Atherosclerosis and Cardiovascular Risk

Thyroid dysfunction has a substantial impact on the formation and progression of atherosclerosis, which is a chronic inflammatory process that forms the basis of numerous cardiovascular illnesses. The complex interaction between thyroid hormones and atherosclerosis involves the regulation of lipid metabolism, inflammatory pathways, and oxidative stress. Thyroid hormones have a crucial impact on lipid metabolism, affecting the concentrations of lipids and lipoproteins in the bloodstream. Hypothyroidism is linked to increased total cholesterol levels and low-density lipoprotein cholesterol (LDL-C), which contribute to developing atherosclerotic lipid profiles [21]. In contrast, hyperthyroidism frequently results in decreased levels of total cholesterol and LDL-C. The changes in lipids affect the beginning and development of atherosclerosis. Thyroid dysfunction not only affects lipid metabolism but also influences inflammatory and oxidative stress pathways, which are essential factors in the development of atherosclerosis. Hypothyroidism is linked to an inflammatory condition defined by elevated levels of inflammatory cytokines and adhesion molecules. The presence of this inflammatory environment encourages the impairment of endothelial function and the attraction of immune cells, which initiates and maintains the development of atherosclerosis [22]. The stability of atherosclerotic plaques plays a crucial role in determining the risk of cardiovascular disease. Thyroid disease affects the stability of plaques through multiple methods. Hypothyroidism has been associated with susceptible plaques distinguished by elevated lipid content, inflammation, and thin fibrous caps. In contrast, hyperthyroidism can result in the formation of calcified plaques that are more solid and less likely to change. Thyroid dysfunction has various consequences on the atherosclerotic process, as evidenced by its impact on plaque stability [22]. The association between thyroid dysfunction and vascular dynamics is a complex interaction that involves various factors such as endothelial function, vascular resistance, blood pressure management, and the development of atherosclerosis. Comprehending these complex interconnections is crucial for deciphering the pathophysiological mechanisms that cause cardiovascular issues related to thyroid disease and for guiding specific treatment approaches.

Clinical manifestations

Thyroid dysfunction substantially affects the cardiovascular system's clinical aspects, leading to symptoms that include alterations in ECGs, abnormalities in cardiac imaging, and critical cardiovascular consequences. This section explores the clinical signs of thyroid dysfunction, emphasizing the complex relationships between thyroid hormones and cardiovascular well-being.

Electrocardiographic Changes

Thyroid dysfunction often leads to electrocardiographic abnormalities. An important discovery is the presence of changes in the shape of the T-wave. Hyperthyroidism can cause an increase in T-wave amplitude, which indicates heightened sympathetic activity and higher cardiac output. In contrast, hypothyroidism is linked to low-voltage T-waves, which frequently suggest reduced heart muscle function [22]. The QT interval on the ECG represents the time it takes for the heart's ventricles to recover and reset after each contraction. A thyroid disorder, specifically hypothyroidism, is associated with the prolonging of the QT interval. Extended restoration of the electrical potential of the heart muscle after each contraction raises the likelihood of abnormal heart rhythms, namely torsades de pointes. This highlights the significance of closely observing the changes in the duration of the QT interval in people with thyroid dysfunction. Thyroid dysfunction is an acknowledged catalyst for arrhythmias, which can lead to disruptions in both atrial and ventricular rhythms. AF is a prevalent abnormal heart rhythm linked to hyperthyroidism caused by an overactive SNS and changes in the electrical activity of the atria [23]. Ventricular arrhythmias, such as ventricular tachycardia, can also happen during prolonged QT intervals.

Conversely, hypothyroidism is associated with bradyarrhythmias, such as sinus bradycardia and atrioventricular (AV) block. Hypothyroidism leads to bradyarrhythmias due to decreased sympathetic activity and poor conduction through the AV node. Thyroid disorders can also cause alterations in the ST segment. Hyperthyroidism may result in ST-segment depression, which could indicate an elevated need for myocardial oxygen. ST-segment elevation can develop in cases of hypothyroidism, commonly accompanied by pericardial effusion and reduced blood flow to the heart muscle [23].

Cardiac Imaging Findings

Echocardiography is a helpful method for evaluating the anatomical and functional characteristics of the heart in the presence of thyroid disease. Hyperthyroidism is linked to attributes of an overactive circulatory system, such as a higher left ventricular ejection fraction (LVEF) and lower systemic vascular resistance. In contrast, hypothyroidism can cause a decrease in LVEF and exhibit characteristics of diastolic dysfunction, emphasizing the effect on the heart's ability to contract and relax [23]. Pericardial effusion can occur as a result of both hyperthyroidism and hypothyroidism. Hyperthyroidism causes an increase in blood volume and a hyperdynamic state, which can result in an increase in cardiac output and potentially lead to pericardial effusion. In contrast, hypothyroidism is linked to the accumulation of mucopolysaccharides in the pericardium, which leads to the development of pericardial effusion and, in extreme instances, cardiac tamponade [24]. The association between thyroid dysfunction and coronary artery disease (CAD) is intricate. Hypothyroidism is an acknowledged condition that increases the risk of atherosclerosis, which might make patients more susceptible to CAD. Nevertheless, in the context of hyperthyroidism, the heightened metabolic demand and hyperdynamic condition might worsen pre-existing CAD or trigger sudden coronary events.

Cardiovascular Outcomes

Thyroid dysfunction is strongly linked to the occurrence of heart failure, a notable cardiovascular consequence. Both hyperthyroidism and hypothyroidism can result in heart failure, although they do so through distinct mechanisms. Hyperthyroidism, characterized by its excessive metabolic activity and heightened need for oxygen in the heart muscle, can result in the development of dilated cardiomyopathy and systolic heart failure. On the other hand, hypothyroidism plays a role in causing problems with the heart's ability to relax and fill with blood during the diastolic phase. This can ultimately lead to heart failure, where the heart is unable to pump blood effectively despite maintaining an average ejection fraction. The correlation between thyroid dysfunction and arrhythmias has significant ramifications for cardiovascular health. AF is a prevalent cardiac rhythm disorder linked to hyperthyroidism, which raises the likelihood of thromboembolic events. Extended QT intervals increase the possibility of ventricular arrhythmias, emphasizing the importance of careful monitoring and prompt management [24]. Thyroid dysfunction can impact the regulation of blood pressure, hence contributing to the development of hypertension. Hyperthyroidism is linked to elevated systolic blood pressure due to heightened sympathetic activity and increased cardiac output.

In contrast, hypothyroidism can cause diastolic hypertension, highlighting the varied effects of thyroid disease on the dynamics of blood pressure. Atherosclerosis, the development and advancement of plaque buildup in the arteries, is a significant cardiovascular consequence in patients with thyroid dysfunction. Hypothyroidism, which is marked by an atherogenic lipid profile and an inflammatory environment, plays a role in speeding up the development of atherosclerosis [24]. On the other hand, hyperthyroidism can worsen existing atherosclerotic processes by increasing the body's need for energy and changing how blood vessels work.

Therapeutic implications

Thyroid Dysfunction Management

The therapeutic approach to thyroid dysfunction is comprehensive, with the goal of normalizing thyroid hormone levels and addressing the complex cardiovascular complications associated with thyroid dysfunction. Thyroid hormone replacement treatment is the fundamental approach to managing hypothyroidism. Levothyroxine is the primary treatment, an artificial version of T4. The objective is to attain and sustain euthyroidism, adjusting the levels of thyroid hormones to restore metabolic balance and alleviate cardiovascular symptoms [24]. The dosage is meticulously adjusted according to the patient's clinical response and regular monitoring of thyroid function tests. Antithyroid medicines are crucial in managing the overproduction of thyroid hormones in hyperthyroidism. Methimazole and propylthiouracil hinder the production of thyroid hormones by disrupting the function of thyroid peroxidase. Medication selection depends on various criteria, including patient preferences, pregnant status, and potential adverse reactions [24]. Efficient management of hyperthyroidism is essential to prevent cardiovascular problems, such as arrhythmias and heart failure. Radioactive iodine (I-131) therapy is a viable option for treating hyperthyroidism, especially when antithyroid medicines are not recommended or not well-received. The thyroid gland selectively absorbs the I-131, causing the death of thyroid tissue and a subsequent decrease in hormone output. Thorough supervision is necessary to make necessary changes to the replenishment of thyroid hormones after therapy [24]. Thyroidectomy is an option for severe hyperthyroidism or when I-131 therapy is not appropriate. Thyroidectomy offers a conclusive resolution but necessitates lifelong administration of thyroid hormone replacement treatment. A thorough evaluation of the hazards and advantages, including possible effects on cardiovascular well-being, is essential in making a decision [25].

Cardiovascular Interventions in Thyroid-Related Heart Disease

Beta-blockers are essential in the management of cardiovascular symptoms related to hyperthyroidism. Beta-blockers alleviate tachycardia, palpitations, and tremors by counteracting the impacts of sympathetic activation. Propranolol, atenolol, and metoprolol are often utilized agents. Nevertheless, it is essential to use caution when administering beta-blockers to individuals with heart failure, as they have the potential to worsen systolic dysfunction. Antiarrhythmic drugs may be recommended for treating arrhythmias that are linked to thyroid disease. Amiodarone, a medication, can be used as a rhythm control strategy for treating AF. When dealing with ventricular arrhythmias, the selection of antiarrhythmic drugs is determined by the particular arrhythmia and the underlying cardiac abnormalities [25]. Diuretics can be used cautiously in instances of heart failure linked to thyroid dysfunction to regulate excessive fluid accumulation and relieve symptoms. Nevertheless, it is crucial to diligently observe the electrolytes and renal function levels to avert any potential consequences. Considering the influence of thyroid dysfunction on lipid metabolism and blood pressure management, it may be necessary to prescribe statins and antihypertensive drugs to control dyslipidemia and hypertension. Statins, such as atorvastatin and simvastatin, can mitigate the atherosclerotic risk linked to hypothyroidism [25]. Antihypertensive medications, such as angiotensin-converting enzyme inhibitors and calcium channel blockers, may regulate blood pressure and keep it within desired levels.

Future directions

Emerging Areas of Research

Precision medicine can customize treatment approaches for individuals with thyroid disease. Comprehending the genetic and molecular factors that influence thyroid function and cardiovascular reactions can facilitate the development of individualized therapeutic strategies. Genetic indicators linked to treatment response and cardiovascular risk could assist clinicians in optimizing therapeutic interventions [25]. Progress in imaging modalities, such as cardiac MRI and PET, provide novel opportunities for evaluating heart anatomy and function in persons with thyroid disease. These modalities offer comprehensive information about the properties of myocardial tissue, blood flow, and ability to survive, which helps identify cardiovascular issues early. Incorporating pharmacogenomics into the management of thyroid dysfunction has the potential to transform treatment regimens significantly. Genetic differences that affect how the body processes drugs and responds to thyroid treatments can be used to develop personalized dose plans, reducing adverse effects and improving treatment results [25].

Potential Therapeutic Targets

Investigating the advancement of thyroid hormone analogs with targeted effects on specific tissues has significant promise for medicinal applications. Focusing on particular pathways within the cardiovascular system and reducing unintended consequences makes it possible to achieve more accurate regulation of the cardiovascular effects caused by thyroid disease. Improvements in procedures for administering I-131 therapy, such as the use of tailored delivery systems or combination therapies, can potentially increase the effectiveness and safety of this therapeutic option. Ongoing research is focused on developing methods to minimize radiation exposure to tissues that are not the intended target and enhance the accuracy of predicting therapy outcomes [26]. Considering the impact of inflammation on the cardiovascular consequences associated with thyroid dysfunction, there is potential for promising new treatments that specifically target inflammatory pathways. Immunomodulatory drugs or therapies that reduce chronic inflammation could provide additional options for avoiding or treating cardiovascular outcomes. The investigation of the possible advantages of nutraceuticals and other treatments in managing thyroid dysfunction and its related cardiovascular effects is a developing field of research. Compounds possessing antioxidant, anti-inflammatory, or vasodilatory characteristics can enhance conventional therapeutic methods and provide synergistic advantages [27]. The area of controlling thyroid disease and its cardiovascular effects is constantly changing. Ongoing research can improve our comprehension of the complex relationship between thyroid function and cardiovascular health by exploring precision medicine, new imaging tools, novel pharmaceutical targets, and innovative therapeutic approaches [28]. These developments may result in more efficient, personalized, and focused strategies for avoiding and treating the cardiovascular problems linked to thyroid disease. Table 2 shows medications used in thyroid disorders and their potential impact on cardiac function.

**Table 2:** Pharmacological interventions and cardiovascular effects

Medication	Cardiovascular effects	Monitoring cardiovascular parameters during treatment
Levothyroxine (T4)	Generally well-tolerated; mimics endogenous thyroid hormone	Regular assessment of heart rate and blood pressure
	May cause transient tachycardia in the early stages of treatment	Electrocardiogram (ECG) to monitor for arrhythmias and QT interval
	Usually does not significantly impact blood pressure	Periodic evaluation of cardiac function, especially in elderly patients
Liothyronine (T3)	Faster onset of action than levothyroxine	Similar monitoring as levothyroxine
	Potential for more pronounced cardiovascular effects	
Antithyroid medications (e.g., methimazole, propylthiouracil)	Methimazole can cause bradycardia and other cardiac effects	Monitoring of heart rate and blood pressure
	Propylthiouracil may be associated with vasculitis and hypertension	Liver function tests, as hepatotoxicity is a rare side effect
Beta-blockers (e.g., propranolol, atenolol)	Manage symptoms of hyperthyroidism such as tachycardia	Continuous monitoring of heart rate and blood pressure during dosage adjustments
	Reduce cardiac workload and improve symptoms of palpitations	Caution in patients with underlying heart conditions; monitor for signs of heart failure or exacerbation of existing conditions
	Can mask tachycardia, so caution in patients with hypothyroidism	
Radioactive iodine (I-131)	Reduces thyroid hormone production by ablating thyroid tissue	Close monitoring of cardiac function, especially in patients with pre-existing cardiovascular conditions
	Potential for transient worsening of hyperthyroidism symptoms	Caution in patients with heart failure or other significant cardiac issues
	Minimal direct cardiovascular effects

## Conclusions

In conclusion, this study elucidates the complex interaction between thyroid disease and cardiovascular well-being. This study reveals essential information about the impact of thyroid hormones on the structure and function of the heart, resulting in clinical symptoms such as arrhythmias and heart failure. Comprehending these intricate systems is crucial for clinical consciousness, timely identification, and focused treatments. The continuous investigation of the connection between the thyroid and the heart shows potential for discovering novel targets for treatment and developing medical methodologies. Ongoing research endeavors strive to enhance the well-being of those affected by thyroid problems by improving the prevention, diagnosis, and treatment of cardiovascular consequences associated with these conditions.
